# Breast cancer risk prediction with a modified BOADICEA model in Danish women

**DOI:** 10.1038/s41416-025-03247-3

**Published:** 2025-11-12

**Authors:** Sif Ingibergsdóttir Novitski, Rikke Louise Jacobsen, Timo Röder, Peter Christoffer Holm, Michael Schwinn, Ilse Vejborg, My Catarina von Euler-Chelpin, Elsebeth Lynge, Sisse R. Ostrowski, Erik Sørensen, Ole Birger Pedersen, Christian Erikstrup, Bitten Aagaard, Henrik Hjalgrim, DBDS Genomic Consortium, Michael Schwinn, Erik Sørensen, Sisse Rye Ostrowski, Ole Birger Pedersen, Christian Erikstrup, Bitten Aagaard Jensen, Henrik Hjalgrim, Thórunn Rafnar, Kari Stefansson, Henrik Ullum, Karina Banasik, Søren Brunak, Thórunn Rafnar, Kari Stefansson, Nasim Mavaddat, Lorenzo Ficorella, Antonis C. Antoniou, Henrik Ullum, Karina Banasik, Søren Brunak, Stig Egil Bojesen

**Affiliations:** 1 Translational Disease Systems Biology, Novo Nordisk Center for Protein Research, Faculty of Health and Medical Sciences, University of Copenhagen, Copenhagen, Denmark; 2 Department of Public Health, University of Copenhagen, Copenhagen, Denmark; 3 Department of Clinical Immunology, Copenhagen University Hospital, Copenhagen, Denmark; 4 Department of Breast Examinations, Copenhagen University Hospital, Herlev Gentofte, Copenhagen, Denmark; 5 Department of Clinical Medicine, Faculty of Health and Medical Sciences, University of Copenhagen, Copenhagen, Denmark; 6 Department of Clinical Immunology, Zealand University Hospital, Køge, Denmark; 7 Department of Clinical Immunology, Aarhus University Hospital, Aarhus, Denmark; 8 Department of Clinical Medicine, Aarhus University, Aarhus, Denmark; 9 Department of Clinical Immunology, Aalborg University Hospital, Aalborg, Denmark; 10 Department of Haematology, Copenhagen University Hospital Rigshospitalet, Copenhagen, Denmark; 11 Department of Epidemiology Research, Statens Serum Institut, Copenhagen, Denmark; 12 Danish Cancer Institute, Danish Cancer Society, Copenhagen, Denmark; 13 deCODE genetics/Amgen, Reykjavik, Iceland; 14 Centre for Cancer Genetic Epidemiology, Department of Public Health and Primary Care, University of Cambridge, Cambridge, United Kingdom; 15 Statens Serum Institute, Copenhagen, Denmark; 16 Department of Obstetrics and Gynaecology, Copenhagen University Hospital Hvidovre, Hvidovre, Denmark; 17 Department of Clinical Biochemistry, Copenhagen University Hospital, Herlev Gentofte, Copenhagen, Denmark

**Keywords:** Risk factors, Predictive markers, Cancer screening

## Abstract

**Background:**

Breast cancer risk prediction approaches clinical practice. The BOADICEA risk model has been updated to consider common breast cancer risk variants, lifestyle/hormonal risk factors and mammographic density (MD).

**Methods:**

49,494 women from the Danish Blood Donor Study were followed for up to 10 years. Modified BOADICEA risks within 5 and 10 years were calculated based on a polygenic breast cancer risk score combined with lifestyle/hormonal risk factors. MD was only known for 4608 women. Calibration was assessed by comparing observed and predicted risks. AUC and Harrell’s concordance index (C-index) were used to assess discriminative ability and sensitivities and specificities were obtained for high and low-risk groups.

**Results:**

Within 5 and 10 years, 367 and 617 women had breast cancer. The 5-year model achieved an AUC of 0.80 (95% CI:0.78–0.81), sensitivity of 0.34 and specificity of 0.92 for all and an AUC of 0.61 (95% CI:0.58–0.65) for the 50-69-year-aged. For this age-group, the sensitivity was 0.46 in the 10-year model. 50% of women with the highest 5-year risk predictions, identified 94.8% of those with incident breast cancers.

**Conclusion:**

The modified BOADICEA risk model provided valid risks among a large retrospective cohort of Danish women.

## Introduction

Breast cancer is the most common cancer in women worldwide [1]. The incidence in Denmark is among the highest globally, with a lifetime risk of developing breast cancer of 15% [2]. Several lifestyle risk factors [3] are known to be associated with breast cancer risk, including late first birth, low parity, exposure to oestrogens, as well as obesity in postmenopausal women and alcohol [4, 5]. Susceptibility to breast cancer is also explained by genetic predisposition, which includes rare high- (*BRCA1* and *BRCA2)* risk variants [6]. In addition, there are more frequent variants that confer intermediate risks [7]. Genome-wide association studies (GWAS) have further identified many common genetic breast cancer risk variants, and combined through Polygenic Risk Scores (PRS), these variants have a substantial influence on a woman’s risk of developing breast cancer [8].

In Denmark, all women 50-69 years of age are invited to undergo biennial mammography screening. Those with known high risk are screened more intensively and excluded from the general population programme. Screening has lowered breast cancer mortality [9–12] but may also result in false-positive mammographic findings, which are benign at follow-up biopsy. A recent study from the US found that 50% of all women will experience a false-positive mammogram over a 10-year period of annual screening and the risk of false-positives increases with screening frequency [13]. In Denmark, the percentage of false positives is much lower, around 2% false positives per invitation round [14]. Of the breast cancers identified by screening, about 5% would not have presented clinically, had the woman not been screened [15]. This overdiagnosis and false positives are costly, have many side effects, and burden the women [16–18].

The current breast cancer screening can be considered a “one-size-fits-all” strategy aiming women at average risk. Recently, models have been developed to provide a personalised risk score for developing breast cancer [19–21]. BOADICEA (Breast and Ovarian Analysis of Disease Incidence and Carrier Estimation Algorithm) is a multifactorial breast cancer risk model that combines a woman’s age, information on rare high- and moderate-risk variants, PRS, breast and other cancer family history, lifestyle/hormonal risk factors and mammographic density into a single absolute risk estimate [20]. This can be used to identify women who may be at increased or decreased risk of developing breast cancer, and who might benefit from risk-stratified screening programmes. The predictive performance of BOADICEA in Danish women is unknown, as are the contributions from each category of risk factors.

To address this, we used BOADICEA, V6 and the largest accessible retrospective cohort data from The Danish Blood Donor Study (DBDS) to test the model’s 5-year and 10-year risk predictions. We included information on lifestyle factors, history of breast cancer, ovarian cancer and pancreatic cancer in the mothers of participants, use of oral contraception and menopausal, hormonal therapy and PRS. For 4,608 women, we had information on mammographic density.

## Methods

### Study population

**Fig. 1:**
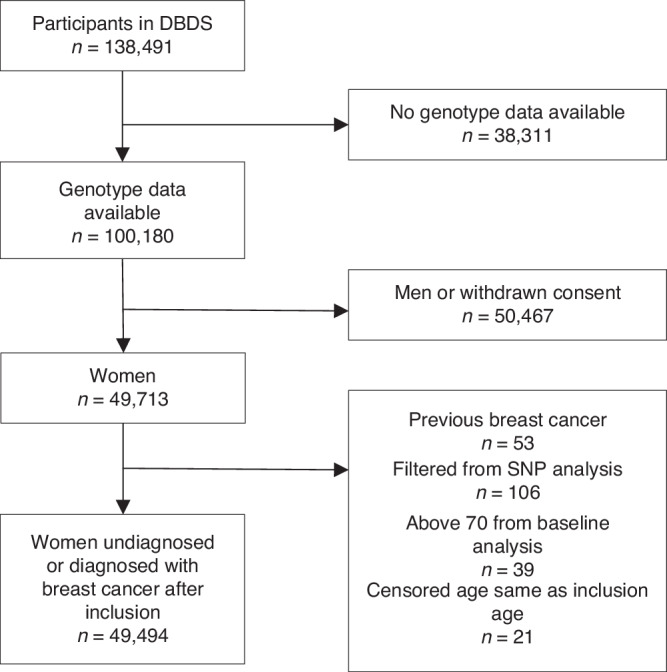
STROBE diagram. Flow chart describing participant inclusions and exclusions applied to define the study cohort.

The Danish Blood Donor Study (DBDS) includes blood donors in the age group 18-70 years, required to be healthy and have a minimum weight of 50 kg [22, 23]. DBDS was initiated in 2010 and is an ongoing nationwide study that covers all blood donation clinics in Denmark. All the blood donors are invited to participate in the study when they are donating blood. They are additionally asked to fill out a questionnaire at the time of enrolment with questions about lifestyle habits and other baseline characteristics. Most DBDS participants have been or are being genotyped (see below). Only participants who had already been genotyped at the time of initiation of the present study (*n* = 100,180) were eligible for inclusion. Genotyped vs. non-genotyped individuals were slightly older and more likely to be men (*p* < 0.001 and *p* < 0.001, data not shown). Participants’ genetic data were linked with nationwide health registers (for an overview of the data used in the study, see Supplementary Fig. S1). The Danish National Patient Registry (NPR) contains information regarding hospital admissions, treatments, operations, and diagnoses. Breast cancer diagnoses were identified using the ICD-10 code C50. Women with one or more breast cancer diagnoses before enrolment were excluded (Fig. 1). Ductal carcinoma in situ and other cancers of the breast were ignored as this is not the target of the BOADICEA model prediction and these tumours constituted 16% of all neoplastic diagnoses from the breast in Denmark in 2024 [24]. All DBDS participants gave informed consent. This study was approved by the National Committee on Health Research Ethics (M-20090237/1-10-72-95-13, NVK-1700407, and SJ-740) and the data protection in the Capital Region of Denmark (P-2019-99).

### Genotyping and PRS calculation

Genotyping of the DBDS cohort was performed by deCODE genetics using the Infinium Global Screening Array from Illumina. The decision to genotype was blind to disease status. The genotyping data were subjected to standard quality control measures and imputation of un-genotyped variants was performed using an in-house reference backbone of North-Western European whole-genome sequences, including approximately 8000 Danish samples, as described elsewhere [23, 25].

The PRS used in the BOADICEA model is based on 313 single-nucleotide variants (SNPs) based on the largest GWAS available to date [8]. Of the 313 SNPs, only 299 were present in the genotype data from DBDS. We excluded SNPs with more than 10% missingness and participants with more than 5% missingness across the 299 SNPs were excluded. Missingness among the remaining SNPs was handled either by replacing the SNP with a proxy variant in high linkage disequilibrium (LD, r^2^ > 0.9) using an in-house Danish LD matrix or by next sampling based on population frequencies. Following these steps, we used a PRS based on 299 SNPs present in the observed or imputed genotype data from DBDS (Supplementary Table S1). The alpha parameter value in BOADICEA, describing the proportion of the overall polygenic variance that is explained by the PRS, was 0.490 [26]. After these steps, the PRS was available for 49,494 women.

PRS_299_ was calculated as the sum of the log odds ratios of all SNPs i for each participant j by the following formula.

1 $${{PRS}}_{J}={\sum }_{i=1}^{299}{n}_{{ij}}\log \left({{OR}}_{i}\right)$$

where n is the number of copies for each SNP in each participant j.

Afterwards, the PRS was normalised based on the cohort included in the study, and the normalised PRS values were used in the model. The association between PRS and breast cancer risk factors was analysed using Spearman’s rank correlation coefficient. We adjusted for multiple testing using the Bonferroni correction and used a significance level of 0.05.

### Risk factors

The date of study baseline was defined as the first date of mammography, the date of questionnaire filled or the date of inclusion in DBDS, whichever came last. This was done to ensure that only data prior to or at baseline was used. Data on age at baseline, age at menarche, age at menopause, height, BMI, and alcohol consumption were extracted from the DBDS questionnaire on the first filled questionnaire from each participant. IQR fencing was used to remove outliers from the questionnaire data. Age at first live birth and parity at the time of study entry were retrieved from the Danish Medical Birth Register (MBR), which records births from 1973 onwards. For women born before 1953, we did not include information from the MBR.

We were able to identify the mothers of 45,125 (91.2%) cohort members via the Danish Civil Registration System and subsequently queried the Danish Cancer Register [27] for relevant cancer diagnoses in these mothers. The registry spans back to 1943 and we queried it for breast cancers, ovarian cancers, and pancreatic cancers in the mothers of study members, using ICD-10 codes C50, C56, and C25, as well as suitable ICD-7 codes for entries before 1978 (Supplementary Table S2). The mother’s age at diagnosis of any of these cancers (as well as at diagnosis of a second breast cancer at least 180 days after a previous one) was used as an input risk factor for BOADICEA (Supplementary Table S3).

The Danish National Prescription Register [28] served as another data source, as we used the redeemed prescription records it contains to determine cohort members’ history of using oral contraceptives and menopausal hormone replacement therapy (HRT) treatments. To increase specificity, two redeemed prescription records were considered a sign of use. We defined current use of oral contraceptives as use of drugs in ATC chapter G03A and the most recent prescription having occurred within two years before study baseline. If prescription records were older than this, we called it the former use of oral contraceptives. Finally, women with fewer than two G03A records (at any time) were determined to have no history of oral contraceptive use. These steps are illustrated in Supplementary Fig. S2. Determining the history of menopausal HRT use followed a similar logic (Supplementary Fig. S3); however, here the period for current use was five years before baseline. We also distinguished between current use of estrogen-only treatments and combination treatments, as these two treatment types are associated with different breast cancer risks. We consulted a study by Danish colleagues [29] to establish which ATC codes should be used for parsing HRT treatment types (Supplementary Table S4). While we did distinguish between former HRT use and absence of HRT use, in practice, this distinction did not add to breast cancer risk in the BOADICEA model used.

Mammographic density as BI-RADS (Breast Imaging Reporting and Data System) score (4th Version) measurements from screening mammograms were only available from the Copenhagen Breast Cancer Screening Programme [30] for 4608 women. For each woman, the mammograms were evaluated independently by two clinicians and given a BI-RADS density score. If the scores differed, the highest one was used as recommended by the American College of Radiology ACR [31]. Owing to the retrospective nature of this study, the mammograms were retrieved from up to six years before baseline, although the majority were from within one year before baseline (Supplementary Figs. S1 and S4).

Importantly, we lacked information on high (e.g., *BRCA1/BRCA2/PALB2*) or intermediate (e.g., *CHEK2/ATM*) risk variants, as there is no central Danish register of previous genetic testing.

### Breast cancer risk calculations

To evaluate the contributions of the different factors, the absolute 5-year and 10-year risks were calculated at baseline using increasing amounts of risk information: (a) age alone, (b) age and PRS, (c) age and risk factors including BI-RADS density, d) age, risk factors, and PRS combined. The BOADICEA breast cancer risk model V6 was used and compared with the observed absolute 5-year and 10-year breast cancer risk. BOADICEA allows for missing input values by using the average population effects over the missing risk factor categories, which is why we included all women, also those without complete information.

### Evaluation of BOADICEA estimates

To assess calibration, women were grouped into deciles according to their calculated risks. For each decile group, the observed risks in percent were estimated using Kaplan-Meier. The start of follow-up (baseline) was the first date of inclusion in DBDS, the first date for the questionnaire filled or the first date of mammography, whichever came last. The end of follow-up was the date of baseline plus 5 or 10 years, the occurrence of breast cancer, death, or the end of the study, whichever came first. For unaffected women with follow-up time shorter than 5 or 10 years, risks were predicted to the age at censoring.

The observed risks were compared to the predicted risks from BOADICEA by inspecting the calibration figures of the observed breast cancer risks versus the predicted mean risk from BOADICEA. To further complement the calibration assessment, we calculated the Brier score as a percentage. The Brier score is a measure comparing the outcome to the predicted risk, where a score of zero is considered the best possible measure.

We evaluated the model’s discriminative ability using Harrel’s C-index and time-dependent AUC. We compared AUCs using a DeLong [32] test (significance level of 0.05) between the model with age and risk factors versus the model with age and PRS, and between the model considering age, PRS, and risk factors versus the two models: age-alone and age-PRS. Confidence intervals of the C-indices were obtained using bootstrapping with 1000 replications.

To investigate the effect of age on AUC, we calculated a series of AUCs by varying the minimum participant age at baseline in comparisons varying from the full cohort (minimum age 18 years) to including only participants aged 60 or older.

Model sensitivity and specificity were calculated for the minimum baseline age groups 18, 20, 30, 40 and 50 using a 5-year breast cancer risk threshold of 1.67% [33, 34], commonly considered to indicate women at increased breast cancer risk. For the 10-year prediction model, a risk threshold of 3.34% was used. Additionally, we applied age-dependent risk thresholds [35] to calculate model sensitivity and specificity for the minimum baseline age groups 40, 45, 50, 55 and 60 for both models.

### Characteristics of low-risk women with breast cancer

From the 5-year model using age, risk factors, and PRS combined, the women with breast cancer during follow-up who were given a low breast cancer risk score (below the median calculated risk) were extracted. Five women without breast cancer per low-risk breast cancer woman were randomly sampled from the entire cohort, matched for age and year at baseline. Mann-Whitney was used to test the significance of the breast cancer risk factors between the low-risk breast cancer women and the control group for the continuous covariates. Bonferroni was applied for correction of multiple testing and a significance level of 0.05 was used. For the categorical variables, Fisher’s exact test was used. We also investigated breast disease history in NPR for these women.

## Results

### Cohort characteristics and PRS association

Eligible women were recruited between 2010–2021. Their median age at baseline was 37 years and the last follow-up date was 2021.08.04. Within 5 and 10 years after baseline, 367 and 617 of the 49,494 women were diagnosed with breast cancer during 236,666 and 386,829 total years of follow-up, respectively. The median follow-up time within 5 years for the entire cohort was 5.0 years (83% complete follow-up) and within 10 years, the median follow-up time was 8.7 years (27% complete follow-up). Within this period, a total of 212 participants died.

The average age at diagnosis was 53 years and age-specific breast cancer incidence rates in our study cohort were slightly higher than the corresponding overall breast cancer incidence rates in Danish women from 2010 to 2021 (Supplementary Fig. S5) and reproducing the expected screening-induced drop for the 55–59-year aged group. The women with known BI-RADS differed from those ≥50-year aged without (Supplementary Table S5), likely mainly due to age difference. None of the breast cancer risk factors examined in the full cohort were associated with PRS_299_ after Bonferroni correction for multiple comparisons (Table 1).

**Table 1:** Breast cancer risk factors and their associations with PRS_299._

	All women (*N* = 49,494)	Women with breast cancer within 10 years after baseline (*N* = 617)	*P*-value: association with PRS_299_
Age at baseline, years	37 (25–50)	50 (43–56)	0.39
Age < 50, *n* (%)	36,889 (74.5)	291 (47.2)	
Year of birth	1975 (1964 – 1987)	1962 (1956–1969)	0.38
Age at menarche, years	13 (12–14)	13 (12–14)	0.96
Missing, *n* (%)	14,296 (28.9)	98 (15.9)	
Age at first live birth, years	27 (24–30)	27 (24–30)	0.27
Not applicable, *n* (%)	21,776 (44.0)	101 (16.4)	
Missing, *n* (%)	2943 (5.9)	97 (15.7)	
Parity			0.04*
0, *n* (%)	21,776 (44.0)	101 (16.4)	
1, *n* (%)	5234 (10.6)	72 (11.7)	
2, *n* (%)	14,107 (28.5)	264 (42.8)	
>2, *n* (%)	5434 (11.0)	83 (13.5)	
Missing, *n* (%)	2943 (5.9)	97 (15.7)	
Height, cm	169 (165–173)	168 (165–172)	0.008*
Missing, *n* (%)	13,263 (26.8)	82 (13.3)	
Body mass index			0.32
<25 kg/m^2^, *n* (%)	22,340 (45.1)	285 (46.2)	
25 - <30 kg/m^2^, *n* (%)	9511 (19.2)	173 (28.0)	
≥30 kg/m^2^, *n* (%)	4095 (8.3)	75 (12.2)	
Missing, *n* (%)	13,548 (27.4)	84 (13.6)	
Oral contraception use			0.12
Current, *n* (%)	19,353 (39.1)	92 (14.9)	
Former, *n* (%)	16,659 (33.7)	236 (38.2)	
Never, *n* (%)	13,482 (27.2)	289 (46.8)	
Missing, *n* (%)	0 (0)	0 (0)	
Alcohol consumption			0.31
0 g/week, *n* (%)	1523 (3.1)	22 (3.6)	
0 ≥ –<5 g/week, *n* (%)	3178 (6.4)	33 (5.3)	
5–<15 g/week, *n* (%)	8973 (18.1)	119 (19.3)	
15–<25 g/week, *n* (%)	1859 (3.8)	33 (5.3)	
Missing, *n* (%)	33,961 (68.6)	410 (66.5)	
Age at menopause, years	50 (46–52)	50 (47–53)	0.15
Missing, *n* (% of women ≥ 50)	5844 (46.3)	129 (39.6)	
Menopausal HRT Use			0.99
Current E-type, *n* (%)	1672 (3.4)	45 (7.3)	
Current other, *n* (%)	2510 (5.1)	47 (7.6)	
Former, *n* (%)	5390 (10.9)	86 (13.9)	
Never, *n* (%)	39,922 (80.7)	439 (71.2)	
Missing, *n* (% of women ≥ 50)	0 (0)	0 (0)	
BI-RADS density			0.07
1, *n* (% of women ≥ 50)	1196 (9.5)	27 (8.3)	
2, *n* (% of women ≥ 50)	1799 (14.3)	50 (15.3)	
3, *n* (% of women ≥ 50)	1322 (10.5)	40 (12.4)	
4, *n* (% of women ≥ 50)	291 (2.3)	7 (2.1)	
Missing, *n* (% of women ≥ 50)	8005 (63.5)	202 (62.0)	
Family History			<0.001
Mothers identified, *n* (%)	45,125 (91.2)	504 (81.7)	
1st breast cancer diagnoses, *n* (%)	2547 (5.1)	76 (12.3)	
2nd breast cancer diagnoses, *n* (%)	36 (0.07)	4 (0.65)	
Ovarian cancer diagnoses, *n* (%)	328 (0.66)	4 (0.65)	
Pancreatic cancer diagnoses, *n* (%)	169 (0.34)	6 (0.97)	
PRS			NA
Median (IQR)	0.0(−0.68–0.67)	0.33 (−0.40–0.91)	
Missing, *n* (%)	0 (0)	0 (0)	

Data are presented as *n* (%) or median (IQR). The association between PRS and risk factors was investigated for all 49,494 women using Spearman’s correlation

coefficient with a significance level (*p* value < 0.05). Prescription history data was converted to ordinal variables for correlation calculations, corresponding to each category’s relative breast cancer risk (oral contraceptive use: current = 3, former = 2, never = 1; menopausal HRT: current other = 3, current E-type = 2, former/never = 1). Family history was converted to 1 for individuals with a history of at least one of the following cancers: first breast cancer, second breast cancer, ovarian cancer, or pancreatic cancer, and 0 for those with no such history, for the association test. Age distributions for the cancer events in study cohort members’ mothers are detailed in Supplementary Table S4. *P values for height and parity are not significant after Bonferroni correction. PRS_299_: polygenic risk score from 299 SNP’s. BI-RADS: Breast imaging and reporting system; a measure of tissue density from the mammograms, 4th Version. *NA* Not applicable.

### Evaluating breast cancer risk predictions

**Fig. 2:**
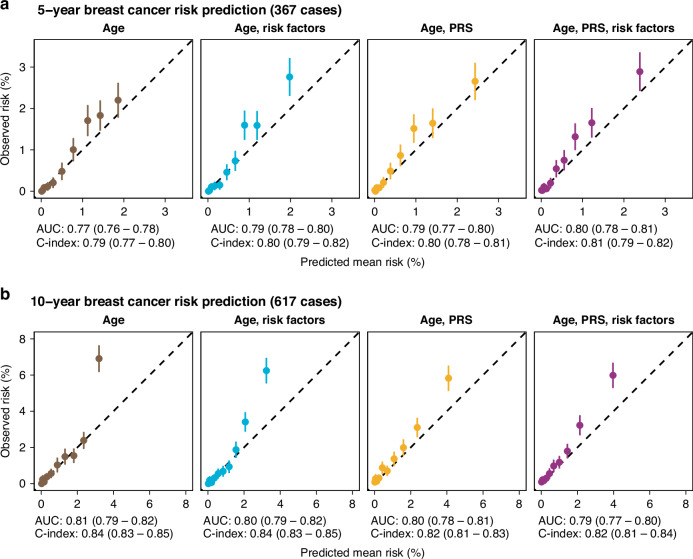
Calibration plots of breast cancer risk prediction in the full cohort of 49,494 women. **a** Predicted risk of breast cancer within 5 years, grouped into deciles and compared with the observed risks. Four different models were evaluated: age alone, age and risk factors, age and PRS, age with both PRS and risk factors. Mean Harrell’s C-index and 95% confidence intervals (bootstrapped with 1,000 iterations) are presented for each model. The dashed line shows the identity function y = x. **b** Same comparisons as above, but for breast cancer risk within 10 years.

In the full cohort, the predicted 10-year and 5-year risks were in line with the observed risks (Fig. 2) for most deciles of predicted risk across the different combinations of information considered in the model. The predicted risks were lower than those observed for the full model for both 5- and 10-years. Inclusion of risk factors improved the model’s discriminative ability (Supplementary Table S6). AUCs and C-indices were around 0.80 for all eight models (two time horizons with four models each). Brier scores were below 5% for all models, indicating that all models were reasonably calibrated (Supplementary Table S6). For the 5-year time horizon, median follow-up was 5.0 (with 25 and 75 percentiles of 5.0–5.0) and 2.4 (1.3–3.7) years for the women without and with breast cancer. For the 10-year time horizon corresponding numbers were 8.8 (6.2–10.0) and 4.16 (2.17–6.34). The normalised PRS distribution was higher for the women with breast cancer (Supplementary Fig. S6).

**Fig. 3:**
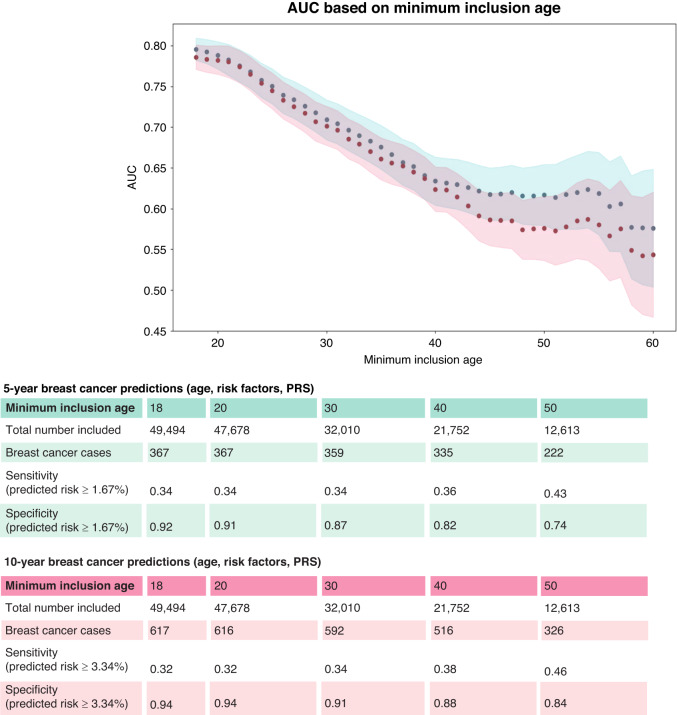
AUC based on minimum baseline age. AUCs were calculated for the full 5-year (blue) and 10-year (red) breast cancer risk models (age, risk factors, PRS) across subsets defined by the minimum study baseline age. The age range considered all women in the cohort until the age of 60. The total number of women included, and the number of breast cancer cases, is listed for both models and minimum baseline age groups of 18, 20, 30, 40, and 50. Sensitivity and specificity were calculated for these groups with risk thresholds of 1.67% (5-year risk) and 3.34% (10-year risk).

As the full cohort contains many young women, we evaluated the performance of the full models (age, PRS, risk factors) when excluding these younger women. In general, AUCs decreased when increasing the minimum baseline age (Fig. 3), with the 5-year model having higher AUCs. Model performance plateaued at the minimum baseline ages of around 40 to 55 years for both the 5-year and 10-year predictions (AUCs around 0.64). Sensitivity increased with higher minimum baseline ages and was slightly higher for the 10-year predictions. The full 10-year model accurately predicted that 46% of the breast cancer cases had a predicted risk of 3.34% or higher in women aged 50 and above. We also evaluated model performance using age-dependent thresholds for high risk, which substantially increased specificity to 0.97 or higher for both models, but with a lower sensitivity over the ages (Supplementary Fig. S7).

When focusing on the 12,613 screening-aged women (ages 50 to 69 years at baseline), we observed large performance improvements when adding PRS and risk factors to the risk model. For this subgroup, the 5-year risk predictions based on the full model (age, PRS, risk factors) had an AUC of 0.62 (0.58–0.65; Supplementary Table S6). Addition of risk factors and PRS improved the 5-year models about equally, while the 10-year model improvements seemed more due to the addition of PRS (Supplementary Table S6). Due to very low numbers of cases (3 and 7 after 5 and 10 years of follow-up) in complete case analyses, we refrained from showing the results of these.

### Low-risk (predicted) women with breast cancer

Since the 5-year risk model was better supported by follow-up time, we used the 5-year time horizon to predict women with low risk, as defined as having a 5-year risk from the full model below the median. Among these 24,747 low-risk women, we identified 19 breast cancer cases, corresponding to 5.2% of all 367 events within 5 years of baseline (Table 2). All affected women were younger than 50 years at the time of baseline, with median ages and cancer diagnosis of 35 and 37 years. When comparing these low-risk cases to matched controls, we did not find any significant differences in any of the risk model input values (Table 2). While the median normalised PRS values were lower (−0.48; IQR = 0.88–0.22, *p* = 0.02) in the low-risk cases than in the controls (0.07; IQR = 0.46–0.67), this difference was not significant after Bonferroni correction. The impact of PRS on overall predicted risk can also be observed from the opposite vantage point: among the 2474 women scoring the highest 5% of PRS values, only 642 (26%) received a low overall predicted risk (below median). None of these 642 women had breast cancer within 5 years of follow-up.

**Table 2:** Characteristics of low-risk women with breast cancer

	Low-risk women (cases) with breast cancer (*N* = 19)	Control group (*N* = 95)	P-value for difference between cases and control
Age at baseline, years	35 (26.5–41)	35 (26–42)	Matched
Age < 50, *n* (%)	19 (100)	95 (100)	
Age at cancer diagnosis, years	37 (30–44.5)		
Missing, *n* (%)	0 (0.0)		
Year of birth	1978 (1971–1987)	1979 (1970.5–1987)	Matched
Missing, *n* (%)	0 (0.0)	0 (0.0)	
Age at menarche, years	13.5 (11.25–14)	13 (12–14)	0.82
Missing, *n* (%)	9 (47.4)	40 (42.1)	
Age at first live birth, years	25 (23–28)	27.5 (26–29)	0.11
Not applicable, *n* (%)	6 (31.6)	45 (47.4)	
Missing, *n* (%)	0 (0)	0 (0)	
Parity			0.08
0, *n* (%)	6 (31.6)	45 (47.4)	
1, *n* (%)	1 (5.3)	14 (14.7)	
2, *n* (%)	9 (47.4)	29 (30.5)	
>2, *n* (%)	3 (15.8)	7 (7.4)	
Missing, *n* (%)	0 (0)	0 (0)	
Height, cm	167.5 (165.25–171.75)	170 (165.5–172.5)	0.56
Missing, *n* (%)	9 (47.4)	40 (42.1)	
Body mass index			0.19
Median (IQR)	27.7 (24.9–30.8)	25.2 (23.1–28.4)	
Missing, *n* (%)	9 (47.4)	40 (42.1)	
Oral contraception use			0.73
Current, *n* (%)	7 (36.8)	47 (49.5)	
Former, *n* (%)	11 (57.9)	32 (33.7)	
Never, *n* (%)	1 (5.3)	16 (16.8)	
Missing, *n* (%)	0 (0)	0 (0)	
Alcohol consumption, g/week			0.36
Median (IQR)	2.57 (1.71–7.71)	6.86 (3.43–8.57)	
Missing, *n* (%)	11 (57.9)	72 (75.8)	
Menopausal HRT Use			0.85
Current E-type, *n* (%)	0 (0)	0 (0)	
Current other, *n* (%)	1 (5.3)	4 (4.2)	
Former, *n* (%)	2 (10.5)	11 (11.6)	
Never, *n* (%)	16 (84.2)	80 (84.2)	
Missing, *n* (% of women ≥ 50)	0 (0)	0 (0)	
Family History			0.19
Mothers identified, *n* (%)	19 (100)	93 (97.9)	
1st breast cancer diagnoses, *n* (%)	0 (0)	7 (7.4)	
Ovarian cancer diagnoses, *n* (%)	0 (0)	1 (1.1)	
PRS			0.02
Median (IQR)	−0.48 (−0.88–0.22)	0.07 (−0.46–0.67)	
Missing, *n* (%)	0 (0)	0 (0)	

Risk factors are listed as median (IQR) or *N* (%). Variables with more than 80% missingness (BI-RADS density, age at menopause) are not presented. There were no diagnoses of a second breast cancer or any pancreatic cancer in the mothers of either the low-risk cases or of the matched controls. Significance between low-risk breast cancer cases and the control group is tested by a Mann-Whitney test. The control group was sampled using age and year at inclusion in the Danish Blood Donor Study. Prescription history data was converted to ordinal variables for the statistical test, corresponding to each category’s relative breast cancer risk (oral contraceptive use: current = 3, former = 2, never = 1; menopausal HRT: current other = 3, current E-type = 2, former/never = 1. Family history was converted to 1 for individuals with a history of either first breast cancer or ovarian cancer, and 0 for those with no such history, for the statistical analysis. The *p*-value threshold for significance after Bonferroni correction is α = 0.05 / 10 = 0.005.

Finally, we checked if the low-risk women with breast cancer had had other breast diseases or symptoms, e.g. carcinoma in situ of the breast, benign mammary dysplasia and others, but no previous breast disease history was recorded.

## Discussion

Personalised breast cancer risk models incorporate the combined effect of genetic and non-genetic risk factors [19, 20]. In this study, we validated the breast cancer risk model BOADICEA with complete information on PRS_299_ for 49,494 women. Where available, information was included on conventional risk factors and around 10% of participants had BI-RADS mammographic density information. This fraction was higher in the screening-age subgroup, which we also evaluated: here, BI-RADS data were available for 36.5% of the 12,613 women in the screening-age subgroup.

Overall, risk model predictions were in line with, but underestimated the observed risks in most deciles of predicted risk. The underestimation may be due to a higher breast cancer incidence in our study cohort compared to the general Danish population, as BOADICEA uses the national (Danish) population rates.

The addition of PRS and risk factors (individually and in combination) to the risk models improved performance in most settings, notably with substantial improvements in the screening-age subgroup. BOADICEA has already been validated in a Dutch [36], Swedish [37] and UK population [38], and very recently in the UK Biobank [39] (Supplementary Table S7 for overview). The UK population study [38] validated the model in women under 50 and found a modest incremental gain in AUC when adding risk factors and PRS, which increased for the screening-age subgroup, similar to our findings. In most settings, PRS had a greater contribution to the model performance compared to risk factors. Overall, our study had slightly higher AUC when considering the full cohort and slightly lower in the screening population.

We analysed the low-risk women with breast cancer using the 5-year model, as we had nearly complete follow-up for this period, making it more well-supported than the 10-year model, for which completeness of follow-up was considerably lower. Under the full 5-year risk model, including age, risk factors, and PRS, risks were generally well-calibrated among women in most deciles of predicted risk. Among the 50% women (n = 24,827) predicted to have low risk, 19 women had the diagnosis of breast cancer within 5 years. All 19 women were below 50 years at baseline and likely not invited to the population screening programme. We found lower PRS values for these women compared to the sampled cohort. Breast cancer among young women below 40 years is rare [40, 41]. Thus, some of these women might have a genetic disposition beyond the PRS_299_ [42], while other cases might be more sporadic [43]. In this study, we do not have any information about mutations in high- or intermediate-penetrant genes such as *BRCA1* or *CHEK2, so* we cannot clarify their contribution. Since such apparently low-risk women will likely be advised to de-escalated screening in future risk-stratified screening programmes, future validation studies of risk models should aim to include a more detailed risk assessment for these women, including full risk factors, family history and rare-genetic variant information.

The concept of personalised prediction models continues to gain relevance [44]. Digitalisation of healthcare systems facilitates the development of personalised preventive and diagnostic tools, and treatments [45]. The freely available CanRisk tool (https://www.canrisk.org/) incorporates BOADICEA and carries a CE-mark [46]. This tool is intended for health care professionals to assist in their communication of breast cancer with patients. In the clinics, this personalised breast cancer risk score could provide information on who would benefit from a shortened or prolonged screening interval. Much focus has been concentrated on identifying women at high risk, but in a population-scale screening programme, identifying women at low risk might be equally, if not more important [21]. Applying age-dependent instead of absolute risk thresholds could enhance specificity and reduce the number of women who should be screened as being at increased risk. This, however, would come with reduced sensitivity. Our results might reassure decision makers when considering risk-stratified breast cancer screening, as the risk predictions seem valid and any de-escalated screening of low-risk women according to the BOADICEA model would likely reduce harms overall.

Our study has several strengths. The study has information on PRS_299_ on almost 50,000 women. Diagnoses and information on mortality are from nationwide registers with high quality [47] and we had no losses to follow up. The AUCs in the overall study were high and likely to be influenced by the wide age range of study participants. As expected, the AUC decreases for higher ages. A less stringent definition of OC and HT use, only requiring one instead of two prescriptions, would increase the number of women with these risk factors, but would likely also increase risk factor misclassification.

The study also has important limitations. Blood donors are generally younger and healthier than the part of the population currently offered breast cancer screening. This potential healthy donor effect [48] did, however, not lead to a lower incidence of breast cancer in the cohort than expected. Furthermore, there may have been some additional selection that could have impacted the results since genotyped individuals were not fully comparable to non-genotyped individuals. The non-inclusion of DCIS and other tumours apart from invasive breast cancer as an outcome might also lead to underestimation of the overall workload from real-life diagnostics in future risk-stratified screening. The collection of risk factor information was not particularly designed for studying breast cancer risk, as illustrated by the high frequency of missingness for some risk variables, particularly mammographic density. Also, we did not have information of cancer family history beyond the mother, nor on mutation status in high- or intermediate-penetrant genes. Information on weight was self-reported, which might limit its predictive value. BOADICEA allows for missing risk factor information and each group of models are based on the same set of individuals, but the missing risk factors might reduce the predictive ability of each model. Any conclusions about the comparative performance of models with different risk factors must be cautious. Another limitation is the fact that the time point for mammographic density measured was not aligned perfectly with the baseline date. This might have reduced prediction accuracy. Since they were only included for 10% of the women, we do not expect the contribution from mammographic density to be the main driver behind the underprediction. Moreover, one measurement of mammographic density predicts its future development quite well [49].

Obviously, to improve generalisability, future studies should include more representative samples, e.g., screening cohorts as in the PRSONAL study [50] or others [51–53] alongside complete follow-up time.

The PRS contributed to model performance in our and in other studies [35, 36, 38]. While the mathematical utility is clear, clinical questions remain to be answered before implementation in the general population screening programmes: (1) Which risk cut-offs and screening interval/modalities produce the optimal outcomes in terms of reduction of advanced breast cancers and breast cancer mortality? (2) How do we best communicate her comprehensive risk estimate and the suggested screening programme to a woman? These and other questions need to be addressed in, preferably, randomised prospective studies in real-life.

In conclusion, we show that risk predictions using BOADICEA were reasonably calibrated among women in Denmark. Low-risk women who developed breast cancer were generally younger and lacked any clearly defined breast cancer risk factors considered in this study, including PRS. BOADICEA provided valid risks among a large retrospective cohort of Danish women.

## Supplementary information

Supplementary material

## Data Availability

The data that support the findings of this study are available from the DBDS, but restrictions apply to the availability of these data, which were used under license for the current study, and so are not publicly available. Data are however available with permission of the DBDS steering committee and the national scientific ethical committee.
