# Supplementary material for: Breast cancer risk prediction with a modified BOADICEA model in Danish women
Source: Br J Cancer. 2025 Nov 12;134(2):259–68. doi: 10.1038/s41416-025-03247-3 (PMC12820242; doi:10.1038/s41416-025-03247-3)
Supplement: Supplementary file 1 — Supplementary material [file 41416_2025_3247_MOESM1_ESM.pdf]

Breast cancer risk prediction with a modified BOADICEA model in Danish women
Supplementary material

Authors:
Sif Ingibergsdóttir Novitski, Rikke Louise Jacobsen, Timo Röder, Peter Christoffer Holm, Michael Schwinn, Ilse Vejborg, My Catarina von Euler-Chelpin, Elsebeth Lynge, Sisse R. Ostrowski, Erik Sørensen, Ole Birger Pedersen, Christian Erikstrup, Bitten Aagaard, Henrik Hjalgrim, DBDS Genomic Consortium, Thorunn Rafnar, Kari Stefansson, Nasim Mavaddat, Lorenzo Ficorella, Antonis C. Antoniou, Henrik Ullum, Karina Banasik, Søren Brunak, Stig Egil Bojesen

Content

Supplementary Figures 2
- Fig. S1: Overview of data sources used in study. 2
- Fig. S2: Scheme for determining history of oral contraception use 3
- Fig. S3: Scheme for determining history of menopausal HRT use 4
- Fig. S4: Time from mammography taken and baseline year 5
- Fig. S5: Breast cancer incidence rates in study cohort and Danish population 6
- Fig. S6: PRS distribution 7
- Fig. S7: Sensitivity and specificity using age-dependent thresholds 8

Supplementary Tables 9
- Table S1: The 299 SNPs, the reference and log odds ratio. 9
- Table S2: ICD codes for identifying mothers' cancer diagnoses in the Danish Cancer Register 25
- Table S3: Cancer diagnosis ages in mothers of study cohort members 25
- Table S4: ATC codes used for determining menopausal HRT prescription history 26
- Table S5: Characteristics of women according to BI-RADS status 27
- Table S6: AUC comparison and brier score 29
- Table S7: Validation studies of breast cancer risk prediction by BOADICEA with PRS 30

Full list of DBDS Genomic Consortium members 31

References 33

Supplementary Figures

Fig. S1: Overview of data sources used in study.

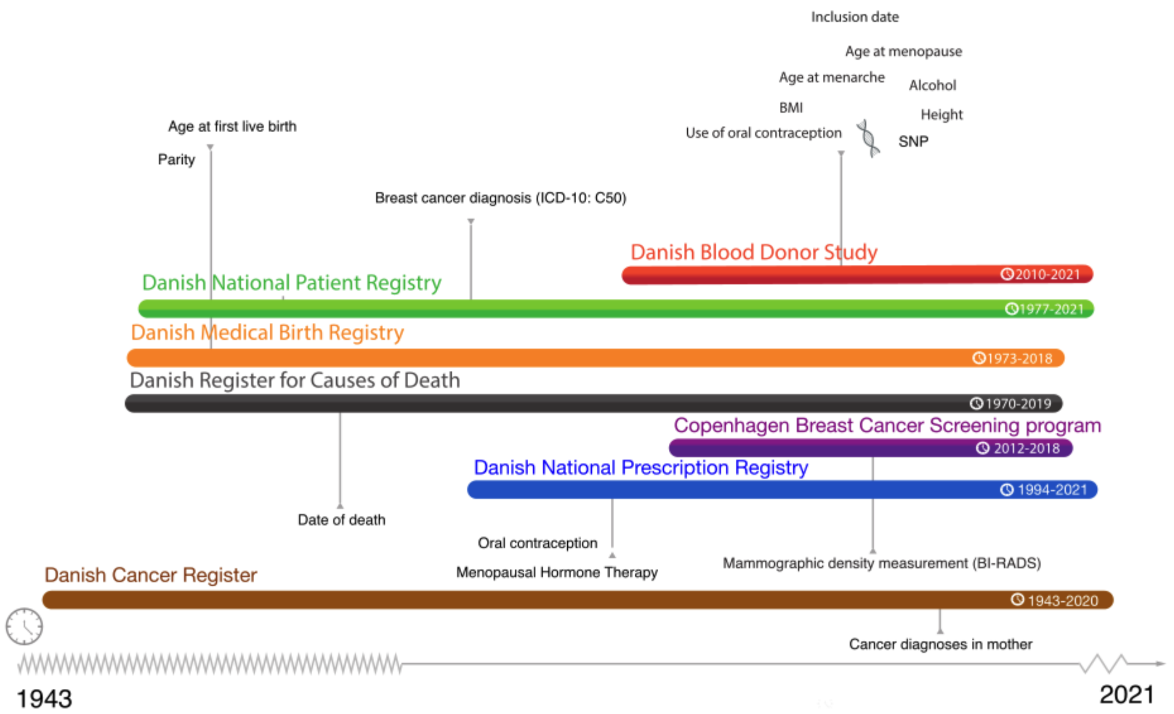

The data sources shown with their time span and variables for the analysis in this study. The registries included are Danish Blood Donor Study (2010 - 2018), Danish National Patient Registry (1977 - 2021), Danish Medical Birth Registry (1973 - 2018), Danish Register for Causes of Death (1970 – 2019), Danish National Prescription Registry (1994 – 2021), and the Danish Cancer Register (1943 – 2020). The database BIRADS (2012 - 2018) was used for extraction of mammographic density for the participants. Cohort members were linked to their mothers via the Danish Civil Registration System (*Det Centrale Personregister).*

Fig. S2: Scheme for determining history of oral contraception use

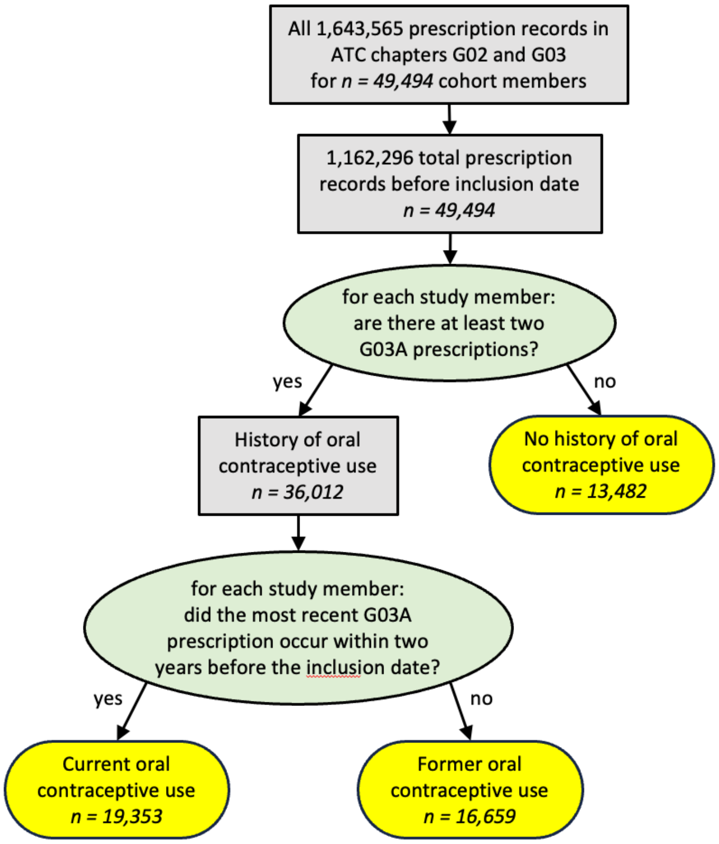

The three categories used as input for breast cancer risk estimation are shown in yellow.

Fig. S3: Scheme for determining history of menopausal HRT use

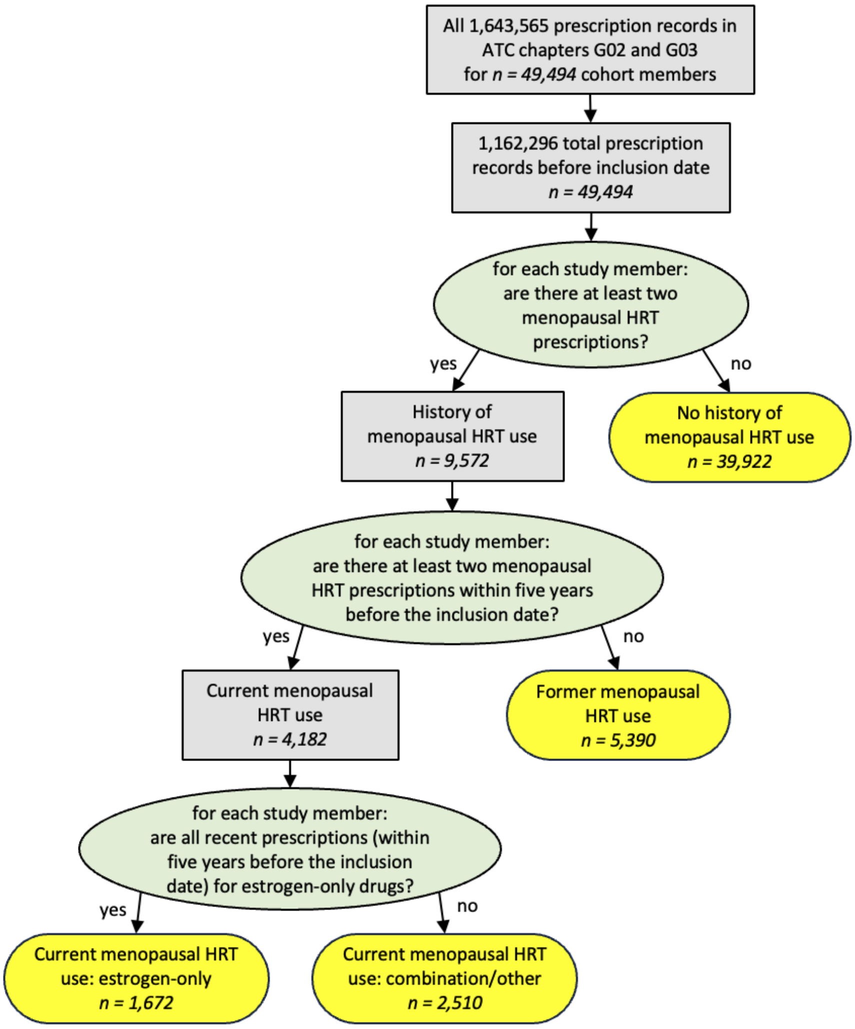

The four categories used as input for breast cancer risk estimation are shown in yellow. ATC codes and corresponding categories for menopausal HRT prescriptions are presented in supplementary table S6.

Fig. S4: Time from mammography taken and baseline year

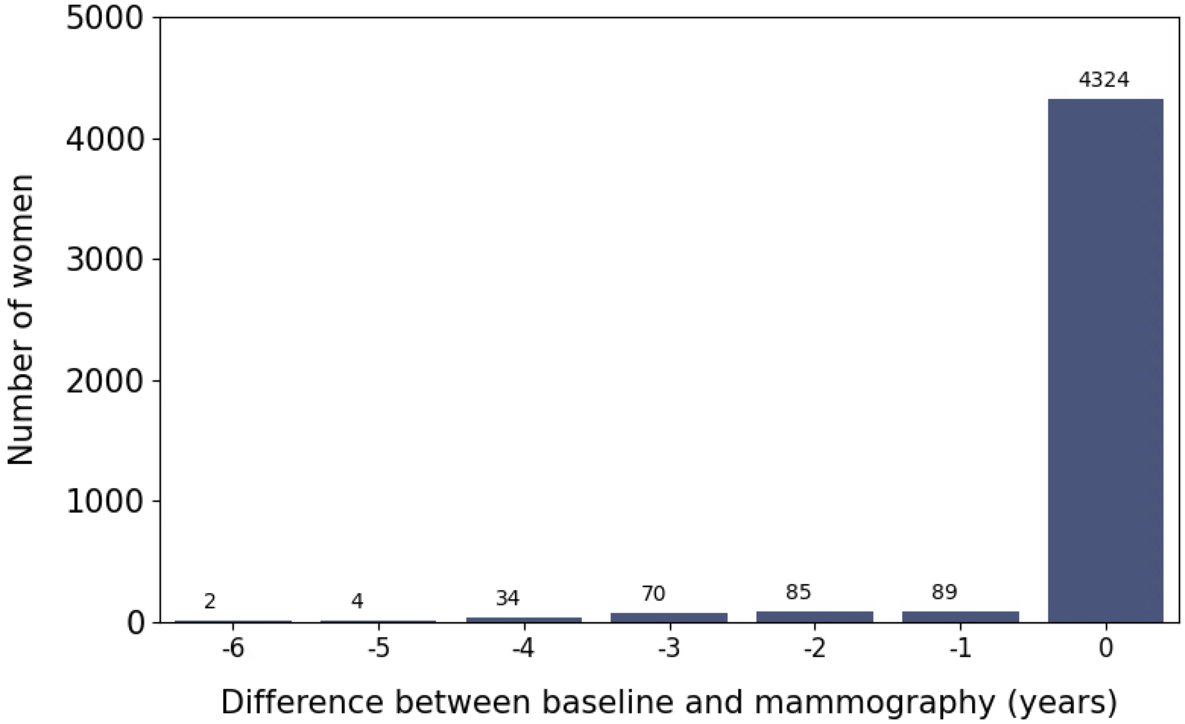

Year 0 is the baseline and the years to the left from baseline indicate how many years before baseline MD was measured.

Fig. S5: Breast cancer incidence rates in study cohort and Danish population

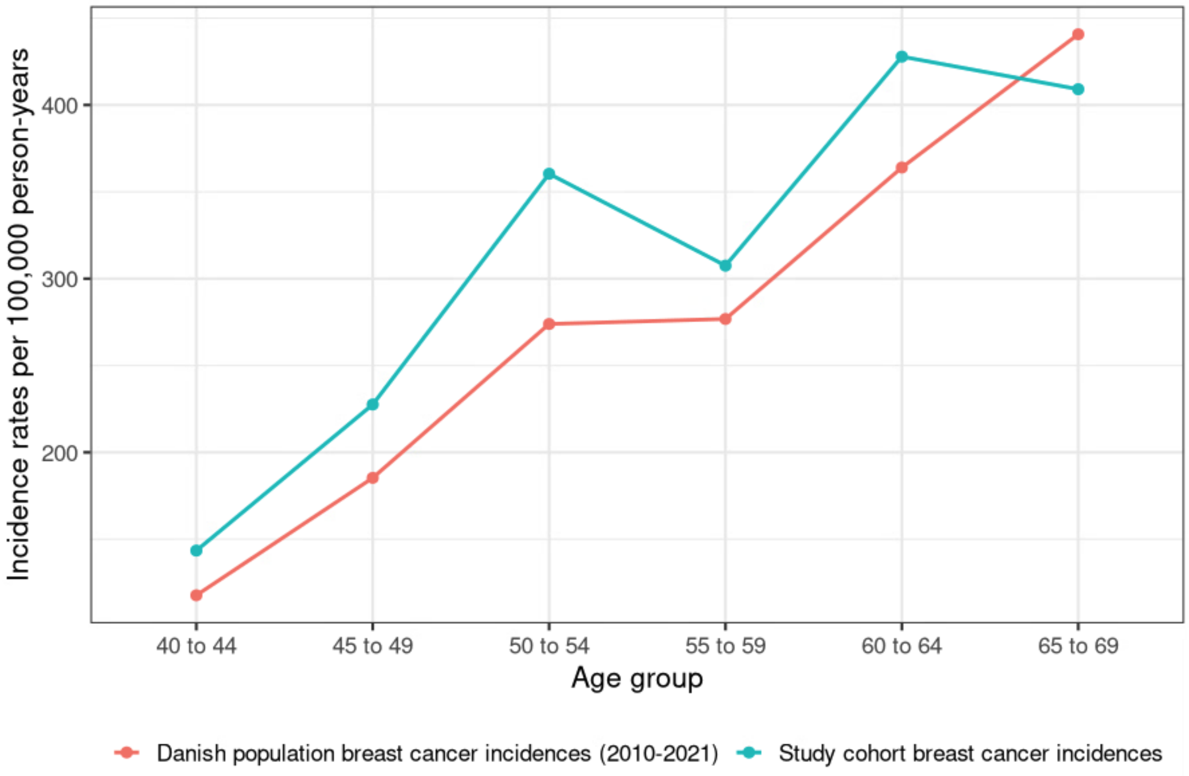

Population data for ages 40-69 and years 2010-2021 taken from NORDCAN:
https://nordcan.iarc.fr/en/dataviz/age_specific?cancers=180&sexes=2&populations=208&mode=cancer&multiple_populations=0&age_span=1&years=2010_2021&multiple_cancers=1&age_end=13&age_start=8&group_years=1

Fig. S6: PRS distribution

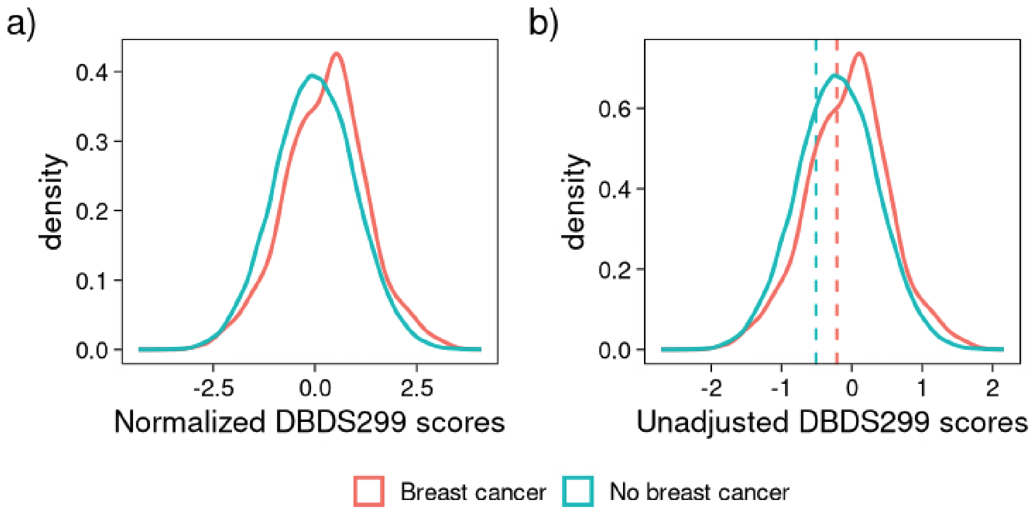

PRS score distribution in 617 women with breast cancer within 10 years of follow-up (red) and in 48,877 women with no breast cancer (blue). a) Normalized scores (mean = 0, s.d. = 1). b) Unadjusted scores. Dashed vertical lines indicate the mean DBDS299 scores for cases and controls in the combined validation set used by Mavaddat et al. (2023; https://doi.org/10.1158/1055-9965.EPI-22-0756 ; also referenced in main text)

Fig. S7: Sensitivity and specificity using age-dependent thresholds

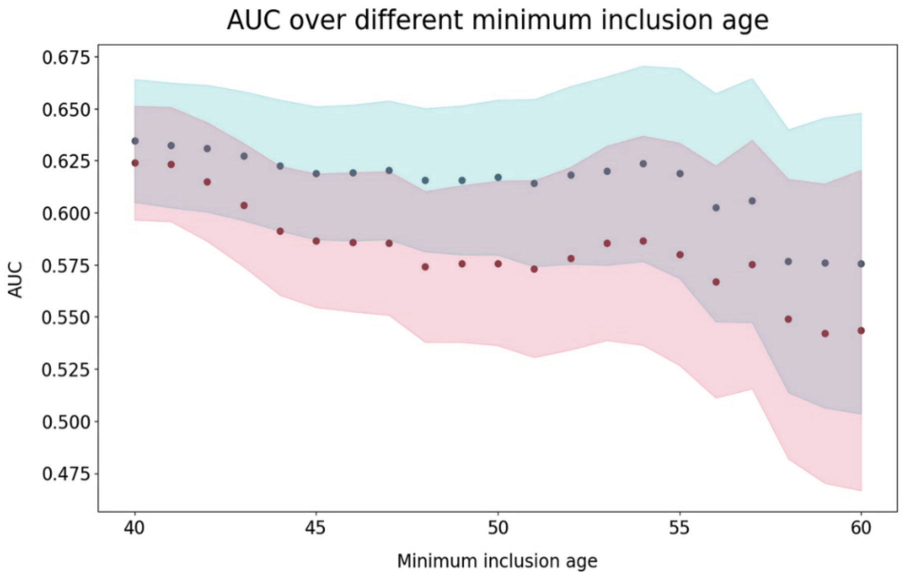

5-year breast cancer predictions (age, risk factors, PRS)

**Minimum inclusion age**	40	45	50	55	60
**High-risk threashold (%)**	1.8	2,4	2.9	3.5	4.3
Total number included	21,752	16,778	12,613	6,782	3,196
Breast cancer cases	335	290	222	127	71
Sensitivity (predicted risk ≥ 1.67%)	0.32	0.21	0.17	0.14	0.07
Specificity (predicted risk ≥ 1.67%)	0.84	0.91	0.93	0.95	0.97

10-year breast cancer predictions (age, risk factors, PRS)

**Minimum inclusion age**	40	45	50	55	60
**High-risk threashold (%)**	3.6	4.8	5.8	7.0	8.6
Total number included	21,752	16,778	12,613	6,782	3,196
Breast cancer cases	516	431	326	192	102
Sensitivity based on predicted risk	0.33	0.19	0.14	0.09	0.04
Specificity based on predicted risk	0.90	0.95	0.97	0.99	0.99

Sensitivity and specificity using age-dependent thresholds from Pashayan N. et al. (1).

Supplementary Tables

Table S1: The 299 SNPs, the reference and log odds ratio.

ID	Reference allele	Effect allele	log odds ratio
18:24518050	AT	A	-0.0599
19:19517054	C	CGGGCG	0.0437
22:40904707	CT	C	0.1148
5:32579616	TCA	T	0.0363
8:17787610	CT	C	-0.0377
17:43212339	C	CT	0.0438
5:44508264	G	GT	-0.1177
1:46670206	TC	T	0.0447
3:49709912	C	CT	-0.0367
1:51467096	CT	C	0.0374
5:52679539	C	CA	0.0571
5:55662540	C	CT	-0.0458
3:55970777	A	AT	-0.1195
3:63887449	T	TTG	0.0648
5:79180995	G	GA	0.0328
5:81512947	TA	T	-0.0598

6:82263549	AAT	A	0.0477
12:83064195	G	GA	0.0671
6:85912194	CAA	C	0.0762
7:91459189	A	ATT	0.0452
14:91751788	TC	T	0.038
4:92594859	TTCTTTC	T	-0.0407
10:95292187	CAA	C	-0.0512
11:108267402	C	CA	-0.0022
9:110303808	TAA	T	0.0797
8:128213561	C	CA	-0.043
3:141112859	CTT	C	0.0551
4:151218296	CATATTT	C	0.0388
1:168171052	CA	C	-0.068
1:204502514	T	TTCTGAAACAGGG	-0.0321
2:217955896	GA	G	-0.2016
rs10012017	G	T	0.0489
rs10069690	C	T	0.0617
rs10074269	T	C	0.0412
rs10096351	A	G	0.0597

rs10120432	T	C	0.0576
rs10164323	C	T	-0.0719
rs10164550	G	A	-0.044
rs1016578	G	A	0.0615
rs10179592	T	C	0.0992
rs10197246	T	C	-0.0492
rs1027113	G	A	0.0647
rs1028016	A	G	-0.0389
rs1036759	G	C	0.0473
rs1061657	T	C	0.0465
rs10764337	A	C	0.0875
rs10796139	G	A	0.0371
rs10816625	A	G	0.1158
rs10832963	T	G	0.0461
rs10838267	G	A	0.0374
rs10862899	C	T	0.0348
rs10885405	C	T	0.0472
rs10896047	G	A	-0.0347
rs10941679	A	G	0.0497

rs10975870	A	G	0.0348
rs10995201	A	G	-0.1345
rs11049431	C	T	-0.0521
rs11065822	G	T	-0.0442
rs11067551	A	G	-0.0428
rs11076805	C	A	-0.03
rs1111207	T	C	0.0346
rs11117758	G	A	-0.044
rs11118563	C	T	0.0418
rs111342015	G	A	-0.064
rs111458676	A	G	-0.0673
rs111833376	C	T	-0.0404
rs111963714	T	G	0.042
rs11205303	T	C	0.0548
rs112476261	C	T	-0.1281
rs11249433	A	G	0.0881
rs112855987	G	A	-0.0134
rs11296	T	C	0.0174
rs114282204	T	C	0.155

rs1154723	T	C	0.0844
rs11624333	T	C	-0.0911
rs11665269	C	T	-0.0415
rs11693806	C	G	-0.0757
rs1172821	C	T	-0.0359
rs117922601	G	T	0.0956
rs11822830	A	G	0.0453
rs11949391	T	C	-0.0564
rs12091730	G	A	0.0499
rs12211970	G	A	0.0326
rs12250948	T	C	-0.0592
rs12287832	C	A	0.0425
rs12406858	A	C	0.0452
rs12422552	G	C	0.0484
rs12449271	T	C	-0.0469
rs12472404	G	C	-0.0066
rs12541094	G	A	0.034
rs12546444	A	T	-0.0745
rs12550713	C	G	0.0642

rs12706954	C	T	-0.0476
rs12709163	C	G	0.0354
rs12870942	T	C	0.0345
rs13039563	G	A	0.044
rs13066793	A	G	-0.0723
rs13147907	A	T	0.0357
rs132289	A	G	-0.1716
rs13244925	A	C	-0.0349
rs13267382	A	G	-0.0417
rs13294895	C	T	0.0653
rs13365225	A	G	-0.076
rs1428387	C	T	0.0944
rs1432679	C	T	-0.0677
rs144767203	A	C	-0.0608
rs149370081	G	A	0.2017
rs150537328	T	C	0.0799
rs1511243	A	G	0.0755
rs1533366	G	T	-0.0391
rs1550622	A	G	0.0593

rs157557	T	C	-0.0363
rs16886165	T	G	0.1366
rs16976596	C	T	-0.0381
rs16991615	G	A	0.076
rs17014016	G	A	0.0352
rs17157372	G	T	-0.0487
rs17268829	T	C	0.0449
rs17343002	G	C	-0.0336
rs17356907	A	G	-0.0867
rs17426269	G	A	0.0494
rs17611291	G	C	-0.0426
rs17625845	T	C	-0.0427
rs17694493	C	G	0.0289
rs17726078	C	G	-0.0473
rs17838698	C	T	0.0592
rs17881320	G	T	-0.0571
rs187010898	C	A	-0.1973
rs187108781	A	G	-0.1101
rs1895062	A	G	-0.0462

rs190884434	T	TA	-0.0435
rs200406386	AAAAG	AAAAGAAAG	-0.0471
rs201415457	A	AAT	-0.0377
rs206966	C	T	0.0516
rs2121348	T	C	-0.0476
rs2151842	C	A	-0.0387
rs2181965	A	G	0.0399
rs2253012	C	T	0.039
rs2277339	T	G	-0.0579
rs2290202	G	T	-0.0589
rs2356656	C	T	0.0316
rs2384736	C	A	0.0404
rs2403907	C	A	-0.0707
rs2454399	T	C	-0.0813
rs2576261	T	G	0.0417
rs2588809	T	C	-0.0474
rs2668667	G	A	-0.054
rs2785646	G	A	-0.0366
rs2787486	A	C	-0.0793

rs2822999	T	G	0.0646
rs2823130	A	G	0.0595
rs28436676	G	A	-0.0898
rs28512361	G	A	0.0736
rs28539243	G	A	0.0477
rs2870876	A	T	0.0469
rs2886671	C	T	-0.0394
rs289997	C	T	-0.0486
rs2992756	T	C	-0.0564
rs3010266	G	A	-0.041
rs310295	C	A	0.0335
rs3215401	A	AG	-0.0549
rs332529	G	A	-0.0564
rs335160	C	A	-0.0386
rs34134147	C	T	0.1405
rs34196306	G	C	-0.0737
rs34872983	G	A	-0.074
rs34914085	C	A	-0.0733
rs35039974	A	T	-0.0423

rs35383942	C	T	0.0917
rs35542655	T	C	0.0477
rs35668161	C	A	0.1147
rs35874463	A	G	0.0782
rs368505905	GT	G	-0.0408
rs371778474	ACC	A	0.1687
rs375193121	CA	C	-0.0391
rs3791977	G	A	-0.0431
rs3819405	C	T	-0.0373
rs3861871	A	G	-0.0382
rs418053	G	C	-0.0553
rs4233486	C	T	0.0426
rs4322799	T	C	-0.0427
rs4399645	T	C	-0.036
rs4439053	G	A	-0.0467
rs4442975	G	T	-0.1318
rs4472923	C	T	-0.0336
rs45631563	A	T	-0.2609
rs45631580	A	G	0.1508

rs4613718	C	T	0.0492
rs4676356	C	A	-0.1232
rs4774565	A	G	-0.0417
rs4784227	C	T	0.107
rs4818836	G	A	0.0946
rs4866496	G	A	0.0391
rs4868701	T	C	0.0363
rs4880038	T	C	0.0249
rs4980029	A	G	0.0762
rs4980386	C	A	-0.0762
rs4983544	T	G	0.0399
rs527616	C	G	0.0455
rs550057	C	T	0.04
rs552647	C	A	0.0748
rs55872725	C	T	-0.0704
rs55910451	A	G	0.047
rs56039025	C	T	-0.0569
rs56069439	C	A	0.0378
rs56387622	T	C	-0.0806

rs56404467	G	A	0.0424
rs56681946	T	C	0.0619
rs5750715	T	A	0.0407
rs58058861	G	A	0.0422
rs5997390	G	A	0.0654
rs6030585	C	G	0.0315
rs60954078	A	G	0.1449
rs610437	T	C	-0.0396
rs612683	A	T	0.0373
rs616488	A	G	-0.0586
rs62255657	C	G	0.0502
rs62329727	T	C	0.1552
rs62331150	G	T	0.0471
rs62334414	C	A	0.0348
rs62485509	G	T	-0.0563
rs625145	A	T	-0.0423
rs62517052	T	C	0.0593
rs62526620	A	G	0.0527
rs630965	C	T	0.0877

rs637868	T	C	0.0385
rs639355	G	A	-0.0376
rs6479868	G	T	0.0472
rs6499648	C	T	-0.0338
rs6596100	C	T	-0.0388
rs6597981	A	G	0.0457
rs661204	G	A	0.1022
rs665889	T	C	0.0361
rs66823261	T	C	0.0477
rs6686987	C	T	-0.0065
rs6725517	A	G	-0.0427
rs6743383	T	A	-0.0331
rs6746250	A	G	-0.0334
rs6756513	G	A	-0.0412
rs6762558	A	G	0.0616
rs68056147	G	A	0.0416
rs6860806	A	G	0.0392
rs6864691	G	A	0.0365
rs6904031	A	T	0.074

rs6913578	A	C	0.0703
rs6940159	T	C	0.0373
rs7072776	A	G	-0.058
rs707475	G	A	-0.0409
rs7121616	A	G	-0.0383
rs7125780	T	G	0.0147
rs7132703	C	T	0.0546
rs71559437	G	A	-0.0568
rs7184573	G	A	-0.0337
rs719338	G	T	-0.0805
rs72658084	C	T	0.1129
rs72755295	A	G	0.1428
rs72931898	G	A	-0.1058
rs7297051	C	T	-0.0856
rs7394715	T	C	-0.0437
rs745570	A	G	-0.0401
rs74765302	G	A	-0.0572
rs7500067	A	G	0.0839
rs7513707	G	A	0.0621

rs7514172	T	A	0.0498
rs75753503	G	T	0.1218
rs76250845	C	T	0.0865
rs77047825	C	G	-0.0748
rs77523078	CAAAAAAA	C	0.0137
rs7800548	T	C	0.0418
rs7830152	A	G	-0.0346
rs78425380	T	C	0.0603
rs7842619	T	G	0.0466
rs78440108	C	T	-0.0577
rs7848334	G	T	0.0153
rs78540526	C	T	0.1782
rs788458	C	T	-0.062
rs7939702	T	G	-0.0543
rs79461387	G	T	-0.0568
rs7971	A	G	-0.0467
rs797736	A	G	0.0401
rs8035987	T	C	-0.0413
rs8042593	G	A	-0.0369

rs8092192	C	G	0.0399
rs851984	G	A	0.0626
rs889310	C	T	0.0394
rs910416	C	T	0.0649
rs9315973	A	G	0.0517
rs9358466	T	C	-0.0321
rs9364472	C	G	-0.0308
rs941764	A	G	0.0513
rs9421410	G	A	-0.0538
rs9611990	C	T	-0.06
rs9693444	A	C	-0.0601
rs9798754	C	T	-0.0367
rs9825432	T	G	-0.0374
rs9882792	C	T	-0.0478
rs9931038	T	C	-0.0211
rs9952980	T	C	-0.0542
rs9954058	G	C	-0.0877

IDs are rsIDs where possible otherwise chr_pos (GRCh37).

Table S2: ICD codes for identifying mothers' cancer diagnoses in the Danish Cancer Register

Cancer type	*ICD-10 code*	*ICD-7 codes (before 1978)*
Breast cancer	C50	**1700**, **1701**, **1702**, **1703**, **1704**, **1705**, 4700, 4701, 4702, 4704, 4705, 6700, 6701, 6702, 7700, 7701, 7702, 7703, 8700, 8701, 8702, 9700, 9701, 9702, 9703, 9704
Ovarian cancer	C56	**1750**, **1751**, **1752**, 1753, **3750**, **4750**, 5750, 5751, 5752, 6750, 6751, 7750, 8750, 9750, 9751, 9752, 9753
Pancreatic cancer	C25	**1570**, **4570**, 5570, 6570, 7570, 8570, 9570

Bolded ICD-7 codes indicate codes which were present in the data.

Table S3: Cancer diagnosis ages in mothers of study cohort members

	All women (N = 49,494)	***Women with breast cancer (N = 617)***
Mother's age at inclusion or death	61 (54 – 70)	70 (63 – 77)
Information available, n (% of N)	45,125 (91.2)	504 (81.7)
Mother's age: 1st breast cancer	55 (48 – 64)	55 (48.8 – 63.2)
Diagnoses, n (% of N)	2,547 (5.1)	76 (12.3)
Mother's age: 2nd breast cancer	61 (50 – 66)	53 (48.5 – 57.2)
Diagnoses, n (% of N)	36 (0.07)	4 (0.65)
Mother's age: ovarian cancer	56 (48 – 66)	55 (54.8 – 59)
Diagnoses, n (% of N)	328 (0.66)	4 (0.65)
Mother's age: pancreatic cancer	66 (56 – 74)	65.5 (59 – 69.8)
Diagnoses, n (% of N)	169 (0.34)	6 (0.97)

Table S4: ATC codes used for determining menopausal HRT prescription history

Category	*ATC codes*	*Details*
Estrogen-only (E)	G03CA03	systemic estrogen, vaginal estrogen tablet, vaginal estrogen ring
	G03CA04	systemic estrogen, vaginal estrogen tablet, vaginal estrogen cream
	G03CA53	systemic estrogen
	G03CA57	systemic estrogen, vaginal estrogen tablet
	G03CB01	vaginal estrogen tablet
Combined estrogen and progestogen (C)	G03FA01	continuous combined
	G03FA11	continuous combined
	G03FA12	continuous combined
	G03FA15	continuous combined
	G03FA17	continuous combined
	G03FB01	cyclic combined
	G03FB05	cyclic combined
	G03FB06	cyclic combined
	G03FB09	cyclic combined
	G03FB11	cyclic combined
	G03HB01	cyclic combined
Other (O)	G02BA03	progestogen IUD
	G03CX01	tibolone
	G03DA02	progestogen alone
	G03DA04	progestogen alone
	G03DB01	progestogen alone
	G03DC02	progestogen alone
	G03DC03	progestogen alone
	G03DC05	tibolone
	G03XC01	raloxifene

Table S5: Characteristics of women according to BI-RADS status

	Women ≥ 50 with known BI-RADS (N = 4,608)	*Women ≥ 50 with unknown BI-RADS (N = 8,005)*	*P-value for difference between women with and without BI-RADS*
Age at baseline, years	54 (51 – 59)	56 (52 – 60)	<0.001
Year of birth	1960 (1954 – 1964)	1957 (1952 – 1960)	<0.001
Age at menarche, years	13 (12 – 14)	13 (13 – 14)	0.12
Missing, n (% of women ≥ 50)	762 (16.5)	2,080 (26.0)	
Age at first live birth, years	27 (24 – 30)	25 (23 – 28)	<0.001
Not applicable, n (% of women ≥ 50)	602 (13.1)	701 (8.8)	
Missing, n (% of women ≥ 50)	856 (18.6)	2,087 (26.1)	
Parity			<0.001
0, n (% of women ≥ 50)	602 (13.1)	701 (8.8)	
1, n (% of women ≥ 50)	673 (14.6)	811 (10.1)	
2, n (% of women ≥ 50)	1,863 (40.4)	3,009 (37.6)	
>2, n (% of women ≥ 50)	614 (13.3)	1,397 (17.3)	
Missing, n (% of women ≥ 50)	856 (18.6)	2,087 (26.1)	
Height, cm	168 (164 – 172)	168 (163 – 171)	<0.001
Missing, n (% of women ≥ 50)	632 (13.7)	1,889 (23.6)	
Body mass index			0.05
<25 kg/m2, n (% of women ≥ 50)	2,285 (49.6)	3,368 (42.1)	
25 - <30 kg/m2, n (% of women ≥ 50)	1,185 (25.7)	1927 (24.1)	
≥30 kg/m2, n (% of women ≥ 50)	476 (10.3)	773 (9.7)	
Missing, n (% of women ≥ 50)	662 (14.4)	1,937 (24.2)	
Oral contraception use			<0.001
Current, n (% of women ≥ 50)	158 (3.4)	213 (2.7)	
Former, n (% of women ≥ 50)	1,678 (36.4)	2,301 (28.7)	
Never, n (% of women ≥ 50)	2,772 (60.2)	5,491 (68.6)	
Missing, n (% of women ≥ 50)	0 (0)	0 (0)	
Alcohol consumption			0.02*
0 g/week, n (% of women ≥ 50)	121 (2.6)	145 (1.8)	
0≥ - <5 g/week, n (% of women ≥ 50)	278 (6.0)	358 (4.5)	
5 - <15 g/week, n (% of women ≥ 50)	864 (18.8)	1,329 (16.6)	
15 - <25 g/week, n (% of women ≥ 50)	223 (4.8)	346 (4.3)	
Missing, n (% of women ≥ 50)	3,122 (67.8)	5,827 (72.8)	
Age at menopause, years	50 (47 – 52)	50 (48 – 53)	<0.001
Missing, n (% of women ≥ 50)	2,571 (55.8)	3,273 (40.9)	
Menopausal HRT Use			0.43
Current E-type, n (% of women ≥ 50)	570 (12.4)	1,002 (12.5)	
Current other, n (% of women ≥ 50)	269 (5.8)	417 (5.2)	
Former, n (% of women ≥ 50)	858 (18.6)	1,206 (15.1)	
Never, n (% of women ≥ 50)	2,911 (63.2)	5,380 (67.2)	
Missing, n (% of women ≥ 50)	0 (0)	0 (0)	
BI-RADS density			
1, n (% of women ≥ 50)	1,196 (26.0)	0 (0)	
2, n (% of women ≥ 50)	1,799 (39.0)	0 (0)	
3, n (% of women ≥ 50)	1,322 (28.7)	0 (0)	
4, n (% of women ≥ 50)	291 (6.3)	0 (0)	
Missing, n (% of women ≥ 50)	0 (0)	8,005 (100)	
Family History			<0.001
Mothers identified, n (% of women ≥ 50)	3,588 (77.9)	5,369 (67.1)	

1st breast cancer diagnoses, n (% of women ≥ 50)	354 (7.7)	460 (5.7)	
2nd breast cancer diagnoses, n (% of women ≥ 50)	6 (0.1)	7 (0.1)	
Ovarian cancer diagnoses, n (% of women ≥ 50)	50 (1.1)	72 (0.9)	
Pancr. cancer diagnoses, n (% of women ≥ 50)	30 (0.7)	66 (0.8)	
PRS			0.68
Median (IQR)	0.0 (-0.67 – 0.67)	0.33 (-0.68 – 0.67)	
Missing, n (% of women ≥ 50)	0 (0)	0 (0)	

Data are presented as n (% of women ≥ 50) or median (IQR). Difference between women with and without BI-RADS are tested by a Mann-Whitney test with a significance level (p value<0.05). Prescription history data was converted to ordinal variables for correlation calculations, corresponding to each category's relative breast cancer risk (oral contraceptive use: current = 3, former = 2, never = 1; menopausal HRT: current other = 3, current E-type = 2, former/never = 1). *P value for alcohol consumption is not significant after Bonferroni correction. PRS_{299}: polygenic risk score from 299 SNP's. BI-RADS: Breast imaging and reporting system; a measure of tissue density from the mammograms 4th Version.

Table S6: AUC comparison and brier score

	5-year prediction (full cohort)	10-year prediction (full cohort)	5-year prediction (50-69 years of age)	10-year prediction (50-69 years of age)
**AUC**				
Age	77.1 (75.8 – 78.3)	80.7 (79.2 – 82.1)	51.0 (47.2 - 54.9)	47.6 (43.8 - 51.5)
Age, Risk factors	79.0 (77.7 – 80.4)	80.2 (78.8 – 81.6)	58.8 (55.0 – 62.6)	51.7 (47.4 – 56.0)
Age, PRS	78.7 (77.4 – 80.0)	79.5 (78.0 – 81.0)	58.1 (54.2 – 62.0)	58.3 (54.4 - 62.1)
Age, PRS, Risk factors	79.6 (78.2 – 80.9)	78.6 (77.1 – 80.1)	61.7 (58.0 – 65.4)	57.6 (53.6 – 61.5)
**AUC comparison, delta AUC (p value)**				
Age, Risk factors vs Age, PRS	0.3 (0.6)	0.7 (0.2)	0.7 (0.8)	-6.6(0.02)
Age, PRS vs Age, PRS, Risk factors	-0.9 (<0.01)*	1.0 (<0.01)*	-3.6 (<0.01)*	0.7 (0.5)
Age vs Age, PRS, Risk factors	-2.5 (<0.01)*	2.1 (<0.01)*	-10.6 (<0.01)*	-10.0 (<0.01)*
**Brier Score (%), 95% CI**				
Age	0.76 (0.68 - 0.84)	1.53 (1.41 - 1.66)	1.79 (1.56 - 2.02)	3.18 (2.80 - 3.56)
Age, Risk factors	0.76 (0.68 - 0.84)	1.53 (1.41 - 1.66)	1.78 (1.55 - 2.01)	3.18 (2.80 - 3.56)
Age, PRS	0.76 (0.68 - 0.84)	1.53 (1.40 - 1.65)	1.78 (1.55 - 2.01)	3.17 (2.79 - 3.55)
Age, PRS, Risk factors	0.76 (0.68 - 0.84)	1.53 (1.40 - 1.66)	1.78 (1.55 - 2.01)	3.17 (2.79 - 3.55)

Table S7: Validation studies of breast cancer risk prediction by BOADICEA with PRS

	**Novitski S et al. 2025, Denmark**		**Lakeman I et al. 2020, Netherlands** (2)	**Choudhury P et al. 2021, UK (3)**	**Yang X et al. 2022, Sweden (4)**		**Petitjean et al. 2025, UK (5)**	
Total N women	49,494		4,377	1337	66,415		217,885	
Subcohort with PRS	49,494		4,377	1337	15,502		217,885	
N Breast cancer								
Within 5 years	347			619	676			
Within 10 years	617		163				6,838	
Average age for full cohort	38		60		57 (unaffected), 59 (bc)		57 (unaffected), 58 (bc)	
Average age for ages <50	32			44				
Average age for ages ≥50	56			59				
**Performance metric**	**C-index**	**AUC**	**C-index**	**AUC**	**C-index**	**AUC**	**C-index**	**AUC**
**5-year breast cancer risk model Full cohort**								
Age	0.79	0.77						
Age, FH				0.69*	0.63	0.61		
Age, risk factors					0.63	0.63		
Age, risk factors, FH, BI-RADS	0.80	0.79						
Age, PRS	0.80	0.79			0.67	0.67		
Age, FH, PRS				0.70*				
Age, risk factors, PRS								
Age, risk factors, FH, PRS				0.70*	0.68	0.68		
Age, risk factors, FH, BI-RADS, PRS	0.81	0.80			0.69	0.69		
**5-year breast cancer risk model Ages ≥50**								
Age		0.51						
Age, FH				0.57				
Age, risk factors								
Age, risk factors, FH, BI-RADS		0.59						
Age, PRS		0.58						
Age, FH, PRS				0.62				
Age, risk factors, PRS								
Age, risk factors, FH, PRS				0.65				
Age, risk factors, FH, BI-RADS, PRS		0.62						
**10-year breast cancer risk model Full cohort**								
Age	0.84	0.81	0.53				0.56	0.56
Age, FH							0.58	0.59
Age, risk factors			0.56				0.57	0.58
Age, risk factors, FH, BI-RADS	0.84	0.80						
Age, PRS	0.82	0.80	0.64				0.64	0.65
Age, FH, PRS								
Age, risk factors, PRS			0.65					
Age, risk factors, FH, PRS							0.65	0.66
Age, risk factors, FH, BI-RADS, PRS	0.82	0.79						
**10-year breast cancer risk model Ages ≥50**								
Age		0.48						
Age, FH								
Age, risk factors								
Age, risk factors, FH, BI-RADS		0.52						
Age, PRS		0.58					0.64	0.65
Age, FH, PRS								
Age, risk factors, PRS								
Age, risk factors, FH, PRS							0.64	0.66
Age, risk factors, FH, BI-RADS, PRS		0.58						

The C-index and AUC is only listed for the sub cohorts with complete information of PRS for more direct overview.
FH: familial history. BC: Breast cancer. *AUCs are listed for the cohort below 50.

Full list of DBDS Genomic Consortium members

PhD Karina Banasik karina.banasik@regionh.dk Department of Obstetrics and Gynaecology, Copenhagen Univesity Hospital, Hvidovre Hospital, Copenhagen, Denmark
PhD Jakob Bay jabay@regionsjaelland.dk Department of Clinical Immunology, Zealand University Hospital, Køge, Denmark
PhD Andrea Barghetti andrea.barghetti@regionh.dk Department of Clinical Immunology, Copenhagen University Hospital, Rigshospitalet, Copenhagen, Denmark
PhD Mette Skou Bendtsen mette.skou.bentsen@regionh.dk Department of Clinical Immunology, Copenhagen University Hospital, Rigshospitalet, Copenhagen, Denmark
MSc Jens Kjærgaard Boldsen jenbol@rm.dk Department of Clinical Immunology, Aarhus University Hospital, Aarhus, Denmark
PhD Søren Brunak soren.brunak@cpr.ku.dk Novo Nordisk Foundation Center for Protein Research, Faculty of Health and Medical Sciences, University of Copenhagen, Copenhagen, Denmark
MD Nanna Brøns nanna.broens@regionh.dk Department of Clinical Immunology, Copenhagen University Hospital, Rigshospitalet, Copenhagen, Denmark
PhD Alfonso Buil Demur alfonso.buil.demur@regionh.dk Institute of Biological Psychiatry, Mental Health Centre, Sct. Hans, Copenhagen University Hospital, Roskilde, Denmark
MD Johan Skov Bundgaard johan.skov.bundgaard@regionh.dk Department of Clinical Immunology, Copenhagen University Hospital, Rigshospitalet, Copenhagen, Denmark
PhD Lea Arregui Nordahl Christoffersen lea.arregui.nordahl.christoffersen@regionh.dk Department of Clinical Immunology, Zealand University Hospital, Køge, Denmark
PhD Maria Didriksen maria.didriksen@regionh.dk Department of Clinical Immunology, Copenhagen University Hospital, Rigshospitalet, Copenhagen, Denmark
PhD Khoa Manh Dinh khoadinh@rm.dk Department of Clinical Immunology, Aarhus University Hospital, Aarhus, Denmark
PhD Joseph Dowsett joseph.dowsett@regionh.dk Department of Clinical Immunology, Copenhagen University Hospital, Rigshospitalet, Copenhagen, Denmark
PhD Christian Erikstrup christian.erikstrup@skejby.rm.dk Department of Clinical Immunology, Aarhus University Hospital, Aarhus, Denmark
MSc Josephine Gladov joglad@rm.dk Department of Clinical Immunology, Aarhus University Hospital, Aarhus, Denmark
PhD Daniel Gudbjartsson Daniel.Gudbjartsson@decode.is deCODE Genetics, Reykjavik, Iceland
PhD Thomas Folkmann Hansen thomas.hansen@regionh.dk Danish Headache Center, Department of Neurology, Copenhagen University Hospital, Rigshospitalet-Glostrup, Copenhagen, Denmark
PhD Dorte Helenius Mikkelsen dorte.helenius.mikkelsen@regionh.dk Institute of Biological Psychiatry, Mental Health Centre, Sct. Hans, Copenhagen University Hospital, Roskilde, Denmark
MSc Lotte Hindhede LOTHIN@rm.dk Department of Clinical Immunology, Aarhus University Hospital, Aarhus, Denmark
PhD Henrik Hjalgrim HHJ@cancer.dk Danish Cancer Society Research Center, Copenhagen, Denmark
PhD Jakob Hjorth von Stemann jakob.hjorth.von.stemann@regionh.dk Department of Clinical Immunology, Copenhagen University Hospital, Rigshospitalet, Copenhagen, Denmark
MD Bitten Aagaard Jensen biaaj@rn.dk Department of Clinical Immunology, Aalborg University Hospital, Aalborg, Denmark
PhD Kathrine Kaspersen kathkasp@rm.dk Department of Clinical Immunology, Aarhus University Hospital, Aarhus, Denmark
MSc Bertram Dalskov Kjerulff berkje@rm.dk Department of Clinical Immunology, Aarhus University Hospital, Aarhus, Denmark
PhD Lisette Kogelman lisette.kogelman@regionh.dk Danish Headache Center, Department of Neurology, Copenhagen University Hospital, Rigshospitalet-Glostrup, Copenhagen, Denmark
PhD Mette Kongstad mette.kongstad.01@regionh.dk Department of Clinical Immunology, Copenhagen University Hospital, Rigshospitalet, Copenhagen, Denmark
PhD Susan Mikkelsen susanmke@rm.dk Department of Clinical Immunology, Aarhus University Hospital, Aarhus, Denmark

MD Christina Mikkelsen christina.mikkelsen@regionh.dk Department of Clinical Immunology, Copenhagen University Hospital, Rigshospitalet, Copenhagen, Denmark
MSc Line Hjorth Sjernholm Nielsen linen3@rm.dk Department of Clinical Immunology, Aarhus University Hospital, Aarhus, Denmark
PhD Janna Nissen ioanna.nissen@regionh.dk Department of Clinical Immunology, Copenhagen University Hospital, Rigshospitalet, Copenhagen, Denmark
PhD Mette Nyegaard nyegaard@hst.aau.dk Department of Health Science and Technology, Faculty of Medicine, Aalborg Univeristy, Aalborg, Denmark
DMSc Sisse Rye Ostrowski sisse.rye.ostrowski@regionh.dk Department of Clinical Immunology, Copenhagen University Hospital, Rigshospitalet, Copenhagen, Denmark
MSc Frederikke Byron Pedersen frederikke.byron.pedersen@regionh.dk Department of Clinical Immunology, Copenhagen University Hospital, Rigshospitalet, Copenhagen, Denmark
PhD Ole Birger Pedersen olbp@regionsjaelland.dk Department of Clinical Immunology, Zealand University Hospital, Køge, Denmark
PhD Liam James Elgaard Quinn liaq@regionsjaelland.dk Department of Clinical Immunology, Zealand University Hospital, Køge, Denmark
PhD Þórunn Rafnar Thorunn.Rafnar@decode.is deCODE Genetics, Reykjavik, Iceland
PhD Palle Duun Rohde palledr@hst.aau.dk Department of Health Science and Technology, Faculty of Medicine, Aalborg Univeristy, Aalborg, Denmark
PhD Klaus Rostgaard klar@cancer.dk Danish Cancer Society Research Center, Copenhagen, Denmark
PhD Andrew Joseph Schork andrew.joseph.schork@regionh.dk Institute of Biological Psychiatry, Mental Health Centre, Sct. Hans, Copenhagen University Hospital, Roskilde, Denmark
PhD Michael Schwinn michael.schwinn@regionh.dk Department of Clinical Immunology, Copenhagen University Hospital, Rigshospitalet, Copenhagen, Denmark
PhD Erik Sørensen Erik.Soerensen@regionh.dk Department of Clinical Immunology, Copenhagen University Hospital, Rigshospitalet, Copenhagen, Denmark
PhD Kari Stefansson kari.stefansson@decode.is deCODE Genetics, Reykjavik, Iceland
PhD Hreinn Stefánsson hreinn.stefansson@decode.is deCODE Genetics, Reykjavik, Iceland
MSc Jacob Træholt jacob.traeholt@regionh.dk Department of Clinical Immunology, Copenhagen University Hospital, Rigshospitalet, Copenhagen, Denmark
PhD Unnur Þorsteinsdóttir Unnur.Thorsteinsdottir@decode.is deCODE Genetics, Reykjavik, Iceland
MD Mie Topholm Bruun mie.topholm.bruun@rsyd.dk Department of Clinical Immunology, Odense University Hospital, Odense, Denmark
PhD Henrik Ullum HEUL@ssi.dk Statens Serum Institut, Copenhagen, Denmark
PhD Thomas Werge thomas.werge@regionh.dk Institute of Biological Psychiatry, Mental Health Centre, Sct. Hans, Copenhagen University Hospital, Roskilde, Denmark
PhD David Westergaard david.westergaard@regionh.dk Department of Obstetrics and Gynaecology, Copenhagen Univesity Hospital, Hvidovre Hospital, Copenhagen, Denmark

References

1. Pashayan N, Antoniou AC, Lee A, Wolfson M, Chiquette J, Eloy L, et al. Should age-dependent absolute risk thresholds be used for risk stratification in risk-stratified breast cancer screening? J Pers Med. 2021 Sep 15;11(9):916.

2. Lakeman IMM, Rodríguez-Girondo M, Lee A, Ruiter R, Stricker BH, Wijnant SRA, et al. Validation of the BOADICEA model and a 313-variant polygenic risk score for breast cancer risk prediction in a Dutch prospective cohort. Genet Med. 2020 Nov;22(11):1803–11.

3. Pal Choudhury P, Brook MN, Hurson AN, Lee A, Mulder CV, Coulson P, et al. Comparative validation of the BOADICEA and Tyrer-Cuzick breast cancer risk models incorporating classical risk factors and polygenic risk in a population-based prospective cohort of women of European ancestry. Breast Cancer Res. 2021 Feb 15;23(1):22.

4. Yang X, Eriksson M, Czene K, Lee A, Leslie G, Lush M, et al. Prospective validation of the BOADICEA multifactorial breast cancer risk prediction model in a large prospective cohort study. J Med Genet. 2022 Dec;59(12):1196–205.

5. Petitjean C, Wilcox N, Ficorella L, Dennis J, Tyrer J, Lush M, et al. Evaluating the performance of the Breast and Ovarian Analysis of Disease Incidence Algorithm model in predicting 10-year breast cancer risks in UK Biobank. J Natl Cancer Inst. 2025 May 1;117(5):948–58.
